# Evaluation of atrial fibrillation using wearable device signals and home blood pressure data in the Michigan Predictive Activity & Clinical Trajectories in Health (MIPACT) Study: A Subgroup Analysis (MIPACT-AFib)

**DOI:** 10.3389/fcvm.2023.1243574

**Published:** 2023-12-20

**Authors:** Aishwarya Pastapur, Nicole A. Pescatore, Nirav Shah, Sachin Kheterpal, Brahmajee K. Nallamothu, Jessica R. Golbus

**Affiliations:** ^1^ Division of Internal Medicine, University of Michigan, Ann Arbor, MI, United States; ^2^ Division of Anesthesiology, University of Michigan, Ann Arbor, MI, United States; ^3^ Division of Cardiovascular Medicine, Department of Internal Medicine, University of Michigan, Ann Arbor, MI, United States; ^4^ Michigan Integrated Center for Health Analytics and Medical Prediction (MiCHAMP), University of Michigan, Ann Arbor, MI, United States; ^5^ The Center for Clinical Management and Research, Ann Arbor VA Medical Center, Ann Arbor, MI, United States

**Keywords:** atrial fibrillation, wearable devices, ambulatory monitoring, irregular heart rate notifications, standardized protocol

## Abstract

**Background:**

The rising adoption of wearable technology increases the potential to identify arrhythmias. However, specificity of these notifications is poorly defined and may cause anxiety and unnecessary resource utilization. Herein, we report results of a follow-up screening protocol for incident atrial fibrillation/flutter (AF) within a large observational digital health study.

**Methods:**

The MIPACT Study enrolled 6,765 adult patients who were provided an Apple Watch and blood pressure (BP) monitors. From March to July 2019, participants were asked to contact the study team for any irregular heart rate (HR) notification. They were assessed using structured questionnaires and asked to provide 6 Apple Watch EKGs. Those with arrhythmias or non-diagnostic EKGs were sent 7-day monitors. The EHR was reviewed after 3 years to determine if participants developed arrhythmias.

**Results:**

86 participants received notifications and met inclusion criteria. Mean age was 50.5 (SD 16.9) years, and 46 (53.3%) were female. Of 76 participants assessed by the study team, 32 (42.1%) reported anxiety surrounding notifications. Of 59 participants who sent at least 1 EKG, 52 (88.1%) were in sinus rhythm, 3 (5.1%) AF, 2 (3.4%) indeterminate, and 2 (3.4%) sinus bradycardia. Cardiac monitor demonstrated AF in 2 of 3 participants with AF on Apple Watch EKGs. 2 contacted their PCPs and were diagnosed with AF. In total, 5 cases of AF were diagnosed with 1 additional case identified during EHR review.

**Conclusion:**

Wearable devices produce alarms that can frequently be anxiety provoking. Research is needed to determine the implications of these alarms and appropriate follow-up.

## 1. Introduction

Atrial fibrillation (AF) is a common arrhythmia associated with increased risk for stroke, heart failure, and mortality (1). The incidence and prevalence of AF are increasing along with AF-associated mortality and hospitalizations (2). Appropriate diagnosis and treatment can decrease AF-related morbidity though this is confounded by the asymptomatic nature of the disease, requiring efforts to improve detection (3).

Adoption of wearable devices (i.e., smartwatches) and home blood pressure (BP) monitors has increased and offers the potential to detect previously undiagnosed arrhythmias such as AF. Studies evaluating the sensitivity and specificity of these devices, however, have demonstrated variable performance and have been limited by their small sample sizes, highly-selective and variable populations, and differing requirements for clinical follow-up to validate test results (4, 5). This is concerning because detection of rhythms such as sinus arrhythmia, premature atrial contractions (PACs), and premature ventricular contractions (PVCs) can also lead to irregular heart rate (HR) notifications (i.e., false positives) which are not due to AF. As wearable device use increases, this has the potential to overwhelm clinician resources.

Herein, we present the results from a secondary analysis of an observational digital health study in which we evaluated the frequency of alarms and then implemented a standardized protocol to address irregular heart rate notifications from wearable devices. In addition to using a rigorous protocol to follow-up on irregular heart rate notifications from either a smartwatch or home BP monitor, we also evaluated participants' electronic health records (EHR) to determine subsequent AF diagnoses in extended 3-year follow-up. We hypothesized that a structured protocol to evaluate irregular heart rate notifications would miss few clinically relevant AF cases.

## 2. Methods

The Michigan Predictive Activity & Clinical Trajectories in Health (MIPACT) study was a prospective observational, digital health study (6). The study enrolled Michigan Medicine patients who were 18 years of age or older, fluent in English, owned an iPhone 6 or newer model, and had regular access to the internet throughout the study period. All participants were provided with an Apple Watch Series 3 or 4, an Omron Evolv Wireless BP Monitor, and the study smartphone application delivered through MyDataHelps. The study was divided into two phases. In both phases, participants were asked to wear their watches for at least 12 h/day for 15 days. Additionally, during the first phase of the study, which lasted up to 45 days, participants were asked to obtain two sets of BP readings daily, with each set consisting of two measurements. During phase two, which lasted until the end of the 3-year study, participants were asked to check at least one set of BP readings each month.

Between 12 March 2019 and 1 July 2019, all participants were provided with an Apple Watch Series 4 and asked during enrollment visits to contact the study team if they received an irregular HR notification through their Apple Watch or BP monitor. These participants were subsequently assessed using structured questionnaires to confirm their comorbidities, evaluate for associated symptoms, and assess anxiety surrounding the notifications with a modified GAD-7 questionnaire. Participants were excluded if they were younger than 22 due to FDA restrictions on use of the Apple Watch ECG feature in these individuals, had a history of AF or stroke, or were on anticoagulation. Participants with concerning symptoms as determined by a study clinician were referred to an urgent care center or to the emergency department as appropriate. Eligible participants were asked to provide 6 ECGs from their Apple Watch, ideally 2 ECGs daily for three days. These were reviewed by a study clinician. Participants with arrhythmias or non-diagnostic ECGs were sent 7-day cardiac monitors. The EHR was later reviewed to determine if participants developed arrhythmias in the greater than 3 years since the study period. Data are summarized as means and standard deviations (SDs) for continuous symmetric variables and as counts and percentages for categorical variables.

## 3. Results

**Figure 1:**
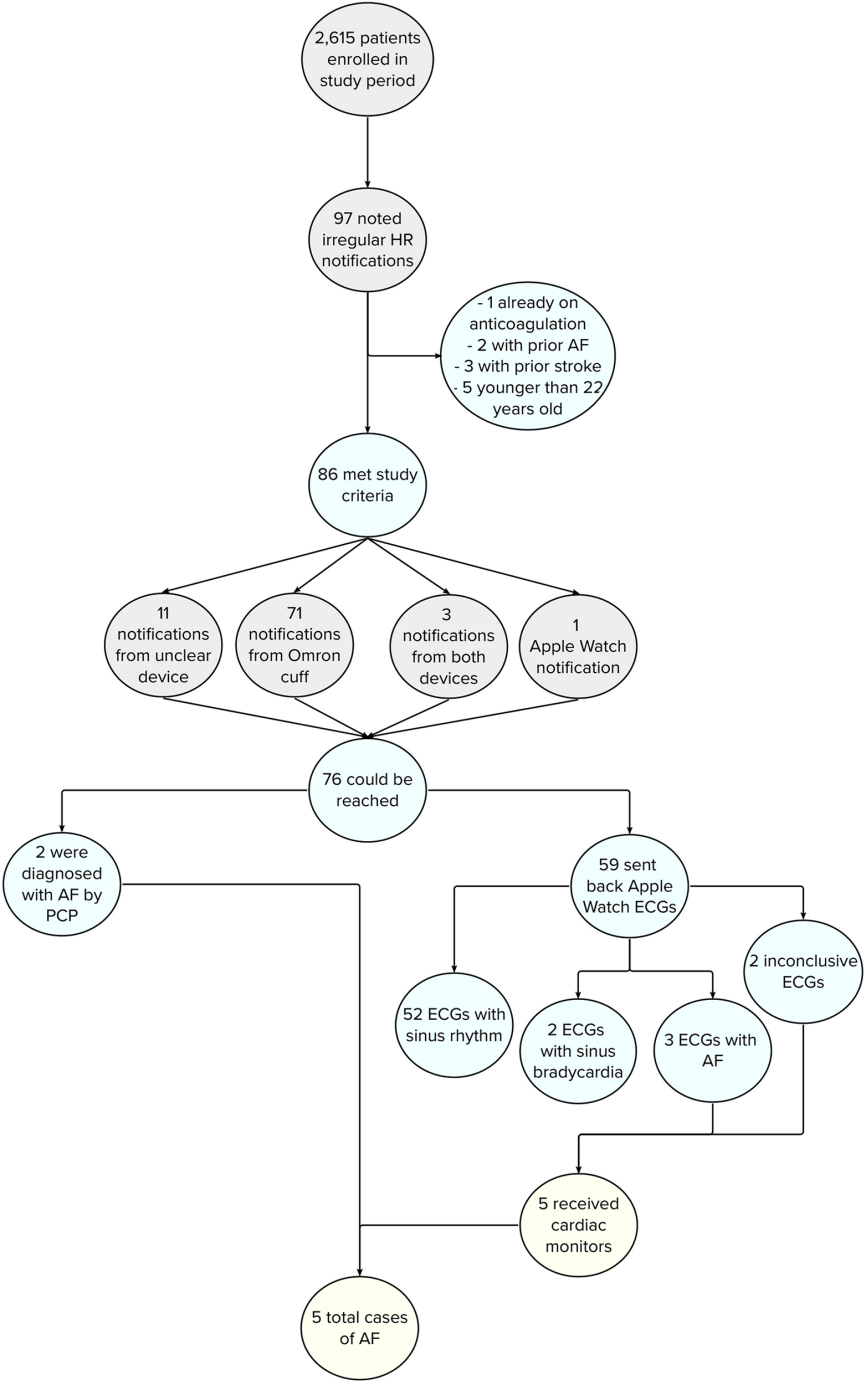
Flowchart depicting incidence of AF in study participants after EHR review. Of the 5 patients who were found to have AF, 4 initially received a notification from their Omron BP cuff and 1 from their Apple Watch.

The MIPACT study enrolled 6,765 eligible participants between 14 August 2018, and 19 December 2019. From 12 March 2019 to 1 July 2019, participants were advised to reach out to the study team for any irregular HR notifications. 2,615 patients were enrolled in this time period (2,435 participants >22 years of age; Supplementary Table S1), of whom 97 (3.7%) contacted the study team for irregular HR notifications. Of these, 5 were younger than 22, 2 had a known history of AF, 3 a previous stroke, and 1 was already on anticoagulation, leaving a final cohort of 86 participants (Figure 1). Participants were 50.5 (SD 16.9) years of age (Supplementary Table S2), and 46 (53.5%) were female. Seventy-one (71; 82.6%) participants received an alarm on the BP monitor, 1 (1.2%) on their Apple Watch, 3 (3.5%) on both devices, and 11 (12.8%) did not recall from which device they received a notification.

Amongst the 86 participants, 76 (89.4%) could be reached and were evaluated by the study team. The mean modified GAD-7 score was 4.27 (SD 4.12), corresponding to no anxiety disorder. However, 26 (34.2%) participants noted mild anxiety surrounding irregular HR notifications and 6 (7.9%) moderate anxiety. Two participants contacted their primary care clinicians after receiving the alarm and were diagnosed with AF. Thus, 74 participants were asked to provide 6 Apple Watch ECGs. Of these participants, 59 (79.7%) sent back at least 1 ECG and 55 (74.3%) 6 ECGs. ECGs revealed sinus rhythm for 52 (88.1%) participants, sinus bradycardia for 2 (3.4%) participants, AF for 3 (5.1%) participants, and inconclusive results for 2 (3.4%) participants (Figure 1). A 7-day cardiac monitor was subsequently mailed to the 5 participants with AF or inconclusive ECGs, confirming AF in 2 of 3 participants with AF noted on their Apple Watch ECGs. In total, 5 (6.6%) cases of AF were diagnosed, 3 by Apple Watch ECGs and 2 by participants' primary care clinicians. In these instances, 4 participants received the initial alarm from the Omron BP cuff and 1 from the Apple Watch.

Three years after the last participant was enrolled, the EHR was reviewed to determine subsequent AF diagnosis. Only 1 additional case of AF was diagnosed in a participant who was initially alerted by an Omron BP cuff and had inconclusive ECGs and a 7-day cardiac monitor revealing supraventricular tachycardia but no AF. Notably, 1 other participant was noted to be in AF on Apple Watch ECG but cardiac monitor revealed only sinus rhythm, and per EHR review, they were not diagnosed with AF on long-term follow-up. An additional 5 participants were found to have other arrhythmias, including ventricular tachycardia, atrial tachycardia, ectopic atrial rhythm, sinus bradycardia, and premature ventricular contractions. In total, 80 irregular heart rate notifications were felt to potentially represent false positive alarms, with 67 related to the Omron BP cuff notification, 4 from both the BP cuff and Apple Watch, and 9 from an unrecalled device. Furthermore, the positive and negative predictive values of our irregular HR alarm follow-up protocol for detecting AF was 100% (3 of 3) and 98.2% (55 of 56), respectively.

## 4. Discussion

Wearable devices and remote monitoring have the potential to increase AF diagnoses. To mitigate this, clinicians need a standardized protocol to address the increasing incidence of irregular HR alarms, which we implemented and evaluated. Of the 2,615 participants enrolled in the study period, nearly 4% experienced alarms. Of 76 participants who could be contacted after reporting an irregular HR by their devices, only 5 were ultimately found to have AF, and only 1 additional participant was diagnosed in the 3 years after the study. This suggests that a standardized algorithm using home-based ECGs coupled with clinician review can help to detect and diagnose AF without overwhelming clinic resources. Additionally, over one third of participants had mild or moderate anxiety surrounding irregular HR notifications. Efficient and structured evaluation of these notifications may help with alarm-associated anxiety though we did not evaluate the impact of our intervention on participant anxiety after implementation of the protocol.

The Apple Heart Study and Fitbit Heart Study revealed that 30%–35% of patients who received irregular HR notifications on their wearable devices had AF on continuous ECG monitoring (7). BP monitors have also been studied for AF detection though results are highly variable especially in light of their evolving capabilities (8, 9). In all prior studies, participants ultimately required clinician follow-up after an irregular HR notification. Follow-up protocols are thus needed to provide guidance on how to address the increasing alarm burden and assuage patient and clinician concerns.

This study has the benefit of long-term follow-up greater than 3 years since the initial study period. However, limitations include limited study period duration and the inclusion of English-speaking patients only. The study also enrolled younger patients and required them to self-report irregular HR notifications. It is also unknown whether the alarms represent false positive alarms or are due to the paroxysmal nature of AF, which can be missed on follow-up in the absence of standardized monitoring. Our study suggests, however, that a standardized protocol using participant obtained ECGs from a smartwatch may be useful for diagnosing AF while minimizing critical resource utilization. With a high positive predictive value, our protocol was generally effective in detecting AF in ambulatory patients who experienced irregular HR notifications, with only 1 case clinically diagnosed after study completion. More notably, our protocol had a high negative predictive value, eliminating the need for further downstream testing for most participants. Additional research is still needed to determine the implications of these alarms and to develop appropriate follow-up protocols, including those that account for participant baseline risk.

## Data Availability

The raw data supporting the conclusions of this article will be made available by the authors, without undue reservation.
