# Supplementary material for: Evaluation of atrial fibrillation using wearable device signals and home blood pressure data in the Michigan Predictive Activity & Clinical Trajectories in Health (MIPACT) Study: A Subgroup Analysis (MIPACT-AFib)
Source: Front Cardiovasc Med. 2023 Dec 20;10:1243574. doi: 10.3389/fcvm.2023.1243574 (PMC10769487; doi:10.3389/fcvm.2023.1243574)
Supplement: Supplementary file 1 [file Table1.docx]

Supplementary Material

Evaluation of Atrial Fibrillation using Wearable Device Signals and Home Blood Pressure Data in the Michigan Predictive Activity & Clinical Trajectories in Health (MIPACT) Study: A Subgroup Analysis (MIPACT-AFib)

Aishwarya Pastapur MD^*^, Nicole A Pescatore MPH, Nirav Shah MD, Sachin Kheterpal MD, MBA, Brahmajee K Nallamothu MD, MPH, Jessica R. Golbus MD, MS

*** Correspondence:** Aishwarya Pastapur MD: [pastapua@med.umich.edu](mailto:pastapua@med.umich.edu)

# Supplementary Data

| **Age Range** | **Number of Participants** |
| --- | --- |
| <40 | 884 |
| 40-64 | 1283 |
| >64 | 448 |
| **Total** | **2615** |

**Supplementary Table 1:** Distribution of ages for full cohort of participants.

| **Age Range** | **Number of Participants** |
| --- | --- |
| <40 | 27 |
| 40-64 | 33 |
| >64 | 26 |
| **Total** | **86** |

**Supplementary Table 2:** Distribution of ages for study participants receiving irregular heart rate notifications who met inclusion criteria.
